# Determinants of default from completion of child immunization among children aged 15–23 months in Kacha Bira district, Kembata Tembaro zone, South Ethiopia: a case–control study

**DOI:** 10.3389/fpubh.2024.1291495

**Published:** 2024-04-12

**Authors:** Sitota Tesema Masebo, Eskinder Wolka, Sintayehu Kussa, Muluken Markos Uloro, Selamu Abera Handiso, Dilugeta Mathewos Libabo, Eyosiyas Abreham Anjajo, Efa Ambaw Bogino

**Affiliations:** ^1^Department of Pediatrics, Shinshicho Primary Hospital, Shishicho, Ethiopia; ^2^School of Public Health, Wolaita Sodo University, Soddo, Ethiopia; ^3^Department of Epidemiology, Wolaita Sodo University, Soddo, Ethiopia; ^4^Department of Internal Medicine, Wolaita Sodo University, Soddo, Ethiopia; ^5^Department of Dermatovenereology, Wolaita Sodo University, Soddo, Ethiopia

**Keywords:** immunization default, determinants, child immunization, children, South Ethiopia

## Abstract

**Background:**

Child immunization is crucial to protect children from vaccine-preventable diseases. However, if a child defaults from completing immunization, they are at a greater risk of contracting such diseases. Previous studies have evaluated various factors that contribute to defaulting from immunization, but they did not consider the fear of COVID-19 as a variable. Additionally, there is inconsistency in the factors identified across different areas. This study aimed to examine the determinants of defaulting from child immunization among children aged 15–23 months in Kacha Bira district, Kembata Tembaro zone, South Ethiopia.

**Methods:**

A study was conducted using a community-based unmatched case–control design to identify the determinants of child immunization completion. The study included 255 children aged 15–23 months in the Kacha Bira district from 3 May 2022 to 1 June 2022, using a multi-stage sampling technique. Face-to-face interviews of mothers or immediate caretakers of the child were conducted using a mobile device, and the questionnaire was developed using the Kobo Toolbox. The data collected were analyzed using SPSS version 25. Multivariable logistic regression was used to identify the determinants, and the adjusted odds ratio with 95% CI and a *p* < 0.05 were considered statistical significant.

**Results:**

The multivariable logistic regression analysis identified four independent predictors of immunization defaulting. Antenatal care (ANC) follow-up [AOR = 5.40, 95% CI (2.24–13.52)], postponing vaccination schedule [AOR = 2.28, 95% CI: (1.05–4.93)], parity of the mother [AOR = 3.25, 95% CI: (1.45–7.27)], and knowledge of the mother about vaccination [AOR = 6.77, 95% CI: (2.33–19.64)] were determinants of immunization defaulting.

**Conclusion:**

In this study, lack of ANC follow-up, postponement of the vaccination schedule, mothers with parity of greater than four, and poor knowledge of the mothers about immunization were identified as determinants of immunization defaulting.

## Background

Immunization is considered one of the most effective and successful public health interventions globally, which helps in reducing childhood morbidity and mortality rates (1). Defaulting on immunization occurs when children miss at least one vaccine dose that is recommended by the National Expanded Immunization Program (EPI) (2). The diphtheria-tetanus-pertussis 3 (DPT3) vaccine is a crucial indicator of immunization completion since it is mainly distributed through horizontal health programs rather than vaccine campaigns (3).

In 2018, ~20 million children worldwide did not receive a complete set of basic vaccines, which include vaccines against tuberculosis (BCG), three doses of the DPT-HepB-Hib vaccine (pentavalent), vaccines against polio, and vaccines against measles (4). In Africa, nearly one in five children miss out on all necessary and basic vaccines (5). In Ethiopia, only 43% of children received all basic vaccinations, and in the Southern Ethiopia region, the percentage is even lower, with only 38% receiving them (6).

The COVID-19 pandemic has had a significant impact on the health system and immunization programs worldwide. As a result, the global coverage of DPT3 vaccines decreased from 86% in 2019 to 83% in 2020. This decrease has led to an increase of 3.4 million completely unvaccinated children, and ~23 million infants did not receive basic vaccines in 2020. This represents the highest number of unvaccinated infants since 2009 (7).

In 1974, the WHO launched the Expanded Program on Immunization (EPI) to ensure the coverage of immunization throughout the world (3). Over the last two decades, vaccine-preventable disease (VPD) surveillance has been conducted in Africa in an integrated manner. All African countries have committed to achieving universal immunization coverage and high-quality surveillance, although this is challenging (5).

In Ethiopia, the Ministry of Health adopted the Expanded Program on Immunization (EPI) in 1980 to reduce morbidity and mortality rates of children with VPDs. Two new approaches, Reaching Every District (RED) and sustainable outreach services (SOS), were introduced in 2003 to enhance immunization coverage and to show progress in the area (8).

Owing to its reliability, cost-effectiveness, and relatively easy administration, EPI is one of the most effective ways to improve health in developing countries. Although there are strategies to overcome the immunization defaulting, it is still a problem. Of the 23 million children who were unvaccinated or under vaccination in 2020, 5.6 million (24%) were defaulters globally. More than 60% of these unvaccinated children are in just 10 countries: India, Nigeria, Congo (DRC), Pakistan, Indonesia, Ethiopia, Brazil, the Philippines, Angola, and Mexico (9).

According to the 2019 mini Ethiopian Demographic and Health Survey (EDHS) data, 76% of children received the first dose of diphtheria, tetanus, and pertussis (DPT) vaccine. However, only 61% of children received all three recommended doses of DPT. This indicates that the magnitude of the immunization default was 15%. Among children aged 12–23 months, the percentage of children who received the first dose of DPT was 72.7%, while only 50.8% received the third dose of DPT, which indicates that the prevalence of immunization defaulting was 21.9% (10).

Defaulting from immunization puts a child at a greater risk of contracting vaccine-preventable diseases (11). Every year, an estimated 2.5 million children under the age of 5 years die due to VPDs worldwide (12). Globally, in 2015, more than 90 million children under the age of 5 years suffered from vaccine-preventable diseases (13). In 2017, more than 17 million cases and 83,439 deaths due to defaulting from immunization were reported worldwide (14). In Africa, over 30 million children under the age of 5 years suffer from VPDs every year because of immunization defaulting, accounting for 33% of the VPD incidence among under-fives worldwide. Recurrent outbreaks of VPD have persisted in many African countries. VPD-related outbreaks tend to occur in areas where low immunization coverage rates are low (5).

When the EPI program was introduced in Ethiopia in 1980, it aimed to fully vaccinate 100% of children less than the age of 2 years by 1990. The target coverage was reset to 75% and the target age group was changed to < 12 months in 1986. However, the program was not successful according to the plan (15, 16).

Previous studies in Ethiopia have identified several factors related to immunization defaulting, including monthly income, number of under-five children, maternal age, parents' educational status, mothers'/caretakers' knowledge about vaccination, and maternal healthcare utilization (17–19).

Previous studies conducted in the Northern region of Ethiopia have shown that certain sociodemographic and economic characteristics, along with maternal knowledge on immunization, place of delivery, and ANC follow-up, can affect the completion of child immunization. However, these studies were carried out in areas where traditional birth attendants were not common, and they were conducted before the COVID-19 pandemic (20, 21).

In the Kacha Bira district, the coverage of the Expanded Program on Immunization (EPI) decreased by 20% in 2019 and 2020. Traditional birth attendants were prevalent in this area, and health facilities were situated at a distance from residential areas. Moreover, there had been no recently published independent research on child immunization in the Southern Ethiopia region. Hence, this study aimed to identify the determinants of defaulting from completion of childhood immunization among children aged 15–23 months in the Kacha Bira district, Southern Ethiopia.

## Materials and methods

### Study design

We employed a community-based unmatched case–control study design.

### Study setting

The study was carried out in the Kacha Bira district, which is situated in the Kembata Tembaro zone in Southern Ethiopia. The district is located 137 km west of Hawassa, the capital of the Southern Nations, Nationalities, and People's Region (SNNPR) and the Sidama Region, and 297 km south of Addis Ababa, the capital of Ethiopia. The district comprises 23 kebeles, with 2 being urban and the remaining 21 rural. According to the Kacha Bira Woreda Health Bureau 2020/21 Report, the estimated total population of the district was 164,382, out of which 21,657 were children under the age of 5 years, and 4,128 were aged between 15 and 23 months. The district has 23 health posts, 6 health centers, and 1 primary hospital, all of which offer immunization services on a routine basis. The study was conducted between 3 May 2022 and 1 June 2022.

### Participants

The source population of the study consisted of all children aged 15–23 months in households located in the Kacha Bira district of the Kembata Tembaro zone in South Ethiopia. The study population, on the other hand, consisted of selected children aged 15–23 months residing in in households located in the chosen kebeles of the Kacha Bira district in the Kembata Tembaro zone. For the purpose of this study, cases were defined as children who had missed at least one recommended immunization dose, while controls were defined as children who had received all recommended immunization doses (20).

### Eligibility criteria

#### Inclusion criteria

Case: All children aged 15–23 months who have received at least one vaccination are included.

Control: All children aged 15–23 months who have received all the recommended vaccinations are included.

#### Exclusion criteria

Children whose parents were too severely ill to respond or who had no full information on the vaccination status of their children were excluded from both cases and controls.

### Sample size determination

We calculated the required sample size using Epi Info software version 7.2.4, with the following parameters: 95% significance, power of 80%, and adjusted odds ratio of 2.33. The case-to-control ratio was 1:2, and the proportion of controls exposed was 48.3%. The odds ratio was taken from a study conducted in the Sodo and Hawasa Zuria districts of Southern Ethiopia (38), taking the mother's educational status as a determinant of default from immunization, resulting in a maximum sample size of 255 (85 cases and 170 controls).

### Sampling procedure and technique

**Figure 1:**
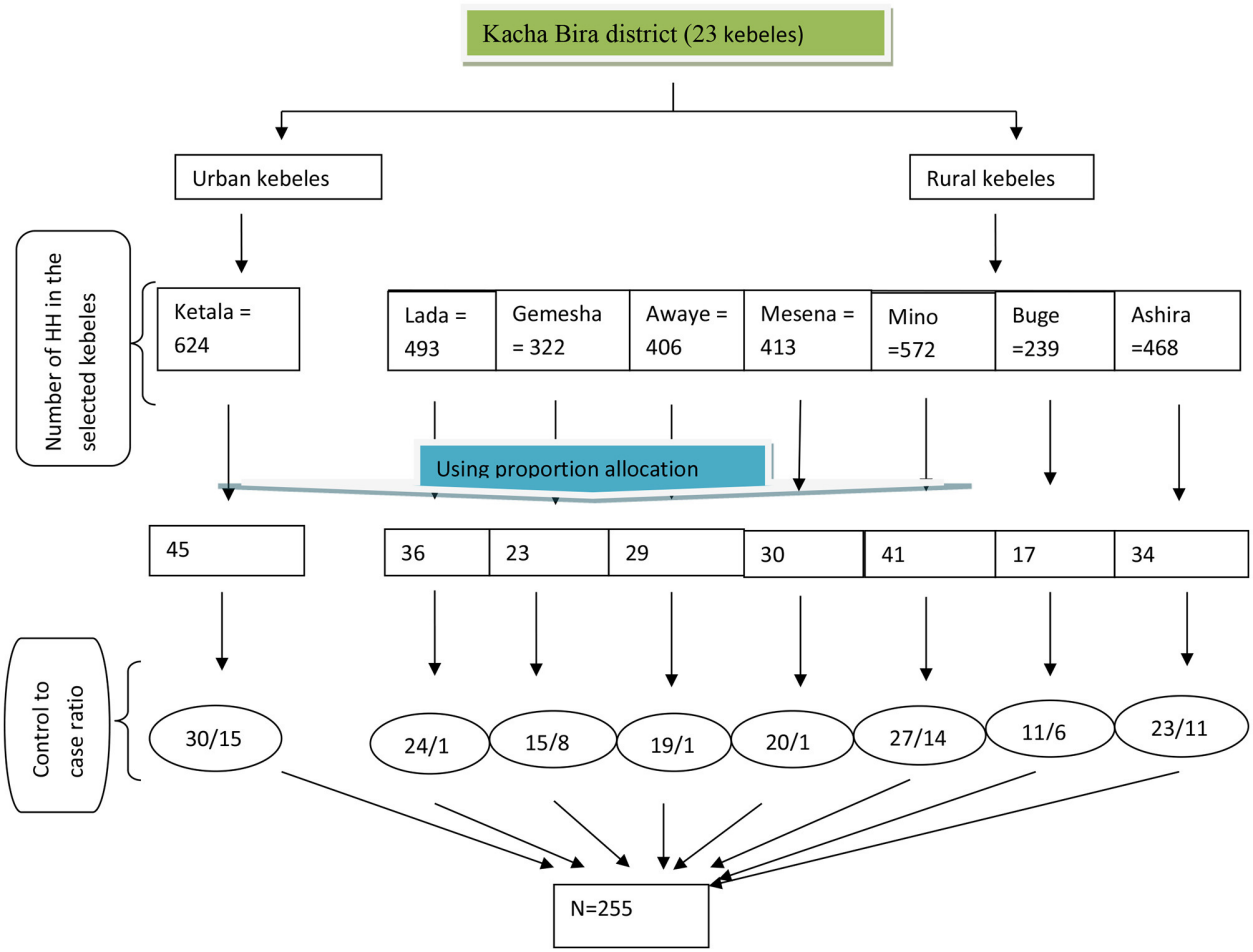
Flowchart: schematic presentation of the sampling technique and procedure to obtain study participants from each selected kebele of Kacha Bira district, Kembata Tambaro zone, South Ethiopia, 2021.

A multi-stage and stratified sampling technique was used to select participants for the study. First, the participants were divided into urban and rural groups. Then, eight kebeles (one urban and seven rural) were chosen using a lottery method from a total of 23 kebeles in the district (two urban and 21 rural). In each selected kebele, a list of cases and controls, along with their complete addresses, were obtained from the health post-EPI registration book. A sampling frame was then prepared from this list. A total sample size of 255 participants (85 cases and 170 controls) was allocated proportionally to each selected kebele. Households with eligible children were chosen through simple random sampling. In households with twins, one child was randomly selected. Tracers were used to locate the selected households. Sampling procedures was shown on Figure 1.

### Variables

#### Dependent variable

➢ Default from completion of immunization (yes/no).

#### Independent variables

➢ **Sociodemographic characteristics:** place of residence, family size, average family income, maternal employment, maternal age, mother's education, and father's education.
➢ **Maternal characteristics:** mother's knowledge, PNC follow-up, ANC follow-up, parity, and tetanus toxoid (TT) vaccination.
➢ **Child characteristics:** sex, birth order, place of birth, and age of the child.
➢ **Health facility-related characteristics:** distance from the health facility, postponing vaccination schedule, waiting time spent in the health facility, and fear of COVID-19 to attend health facilities.

#### Operational definitions (work definition)

**Defaulting from completion of immunization:** A child aged between 15 and 23 months who had missed at least one dose of the recommended routine vaccination schedule at the time of data collection (20).

**Completion of immunization:** A child between 15 and 23 months of age who had received all recommended routine vaccinations at the time of data collection (20).

**Knowledge of mothers about immunization:** Mothers' awareness of the schedule and importance of immunization. This was assessed using eight immunization-related yes/no knowledge questions. The mothers who answered four or more questions were considered to have good knowledge and those below were considered to have poor knowledge (22).

**Immunization status:** Being fully/partially vaccinated or unvaccinated (22).

### Measurement

An electronic data collection system using Kobo Collect version 2021.2.4 software was utilized to collect data. The questionnaire was developed by reviewing relevant literature. To ensure the completeness of data, face-to-face interviews were conducted with the mother or the immediate caretaker of the child. Additionally, the immunization certificate was obtained, if available. The vaccination status was determined by obtaining vaccination cards, vaccination history, or both from the mother or the caretaker.

The study participants were assigned identification numbers, which were used to distinguish between cases and controls. The identification number of the supervisor was used to determine whether a participant was a case or a control before data collection. To ensure unbiased data collection, the data collectors were intentionally kept blind to the status of the respondents, preventing them from identifying the participants as cases or controls.

### Data quality management

The survey was first prepared in English and later translated into Amharic. To ensure consistency, the Amharic version of the questionnaire was then translated back into English. The data were collected by two nurses and one supervisor, all of whom received 2 days of training from the principal investigator. The data collectors were nurses with diplomas, while the supervisor held a BSc in Nursing. Before the actual data collection began, a pre-test was conducted on 5% of the sample, consisting of 13 participants (eight controls and five cases) outside the study area. Throughout the data collection process, the supervisor checked for completeness and consistency of the collected data.

### Statistical method

The data were collected, entered into the electronic system, and then downloaded and exported to SPSS version 25 for statistical analysis. Before the analysis, the data were arranged, edited, and cleaned by running simple frequencies and cross-tabulations. Distributional plots, tests, and categorization of quantitative variables were performed. The distribution of the continuous variables was checked for normality. Means with standard deviations and medians with interquartile ranges were used to summarize the normally and non-normally distributed continuous variables, respectively. The bivariate logistic regression analysis was used to determine the association between the explanatory and outcome variables. This was followed by a multivariable logistic regression analysis using those variables with a *p*-value of 0.2 or less in the bivariable analysis. The goodness of fit of the statistical model was checked using the Hosmer–Lemeshow test. Multicollinearity was assessed using a tolerance test and a variance inflation factor. Odds ratios (ORs) with 95% confidence intervals were used to measure the strength between dependent and independent variables. Statistical significance was set at a level of 0.05.

## Results

### Sociodemographic characteristics of the respondents

Out of 255 children aged between 15 and 23 months, 249 caretakers, including 83 cases and 166 controls, were interviewed. The response rate for this study was 97.6%. Most primary caretakers for both cases (69, 83.1%) and controls (150, 90.4%) were mothers. The educational background of care givers evaluated. Then, 39 (47%) of mothers among and 65 (39.6%) among controls group attended primary school (grade 1 to 8) while 18 (21.7%) of mothers among cases and 39 (23.8%) among controls completed secondary education (grade 9 to 12). Sociodemographic characteristics are shown in Table 1.

**Table 1:** Socio demographic characteristics of the respondents at Kacha Birra district, kembata Tembaro zone, south Ethiopia, 2022.

**Variables**	**Category**	**Immunization default**	
		**Cases [No. (%)]**	**Controls [No. (%)]**
Primary care takers	Mother	69 (83.1)	150 (90.4)
	Father	9 (10.8)	10 (6.0)
	Others	5 (6.1)	6 (3.6)
Maternal age	≤ 24 years	15 (18.3)	27 (17.0)
	25–34 years	50 (61.0)	89 (56.0)
	≥35 years	17 (20.7)	43 (27.0)
Father's age	≤ 24 years	6 (7.9)	20 (12.6)
	25–34 years	19 (25.0)	46 (28.9)
	≥35 years	51 (67.1)	93 (58.5)
Marital status	Married	67 (80.7)	143 (86.1)
	Divorced	5 (6.1)	9 (5.4)
	Widowed	4 (4.8)	6 (3.6)
	Single	7 (8.4)	8 (4.9)
Religion	Orthodox	12 (14.7)	30 (18.1)
	Protestant	60 (72.3)	117 (70.5)
	Catholic	9 (10.8)	15 (9.0)
	Muslim	1 (1.2)	3 (1.8)
	Others	1 (1.2)	1 (0.6)
Family size	≤ 5	37 (44.6)	75 (45.2)
	>5	46 (55.4)	91 (54.8)
Monthly income	≤ 1,000 ETB	13 (15.7)	30 (18.1)
	1,001–3,000 ETB	42 (50.6)	78 (47.0)
	>3,000 ETB	28 (33.7)	58 (34.9)
Place of residence	Rural	69 (83.1)	137 (82.5)
	Urban	14 (16.9)	29 (17.5)
Mother's educational status	Illiterate	17 (20.5)	24 (14.6)
	Primary school (1–8)	39 (47.0)	65 (39.6)
	Secondary school (9–12)	18 (21.7)	39 (23.8)
	College and above	9 (10.8)	36 (22.0)
Mother's occupation	Housewife	54 (65.1)	95 (58.3)
	Government employee	8 (9.6)	29 (17.8)
	Merchant	17 (20.5)	28 (17.2)
	Other	4 (4.8)	11 (6.7)
Father's educational status	Illiterate	8 (9.6)	18 (10.8)
	Primary school	15 (18.1)	35 (21.1)
	Secondary school	31 (37.3)	64 (38.6)
	College and above	19 (22.9)	44 (26.5)
Father's occupational status	Government employee	17 (20.5)	38 (22.9)
	Merchant	18 (21.7)	58 (34.9)
	Farmer	35 (42.2)	60 (36.1)
	Other	2 (2.4)	2 (1.2)

### Maternal and child-related characteristics

The average age of the children was 19.1 months, with a standard deviation of 2.47. Most cases (85.5%) and controls (97.6%) were born in a health institution. During data collection, 64 (77.1%) cases and 148 (89.2%) controls had vaccination cards. Among respondents, 53 (63.9%) cases and 149 (89.8%) controls reported having regular antenatal care (ANC) follow-ups and receiving tetanus toxoid (TT) vaccination during their follow-ups. Furthermore, 49 (66%) mothers categorized as cases and 126 (80.8%) categorized as controls reported having a parity of less than four. Maternal and child-related factors are listed in Table 2.

**Table 2:** Maternal and child related characteristics of the respondents at Kacha Birra district, kembata Tembaro zone, south Ethiopia, 2022.

**Variables**	**Category**	**Immunization default**	
		**Cases [No. (%)]**	**Controls [No. (%)]**
Sex of the child	Male	39 (47.0)	85 (51.2)
	Female	44 (53.0)	81 (48.8)
Place of birth	Home	12 (14.5)	4 (2.4)
	Health institution	71 (85.5)	162 (97.6)
Age of the child	15–17 months	27 (32.5)	47 (28.3)
	18–20 months	30 (36.1)	65 (39.2)
	21–23 months	26 (31.3)	54 (32.5)
Birth order of the child	1st−3rd	48 (57.8)	102 (61.4)
	4^th^ and above	35 (42.2)	64 (38.6)
Availability of vaccination card	Yes	64 (77.1)	148 (89.2)
	No	19 (22.9)	18 (10.8)
ANC follow up	Yes	53 (63.9)	149 (89.8)
	No	30 (36.1)	17 (10.2)
Number of ANC visits	≤ 2 visits	12 (22.6)	39 (26.2)
	>2 visits	41 (77.4)	110 (73.8)
TT immunization	Yes	50 (60.2)	132 (79.5)
	No	33 (39.8)	34 (20.5)
PNC follow up	Yes	33 (39.8)	118 (71.1)
	No	50 (60.2)	48 (28.9)
Parity	≤ 4	49 (66.2)	126 (80.8)
	>4	25 (33.8)	30 (19.2)

### Knowledge of mothers regarding immunization

Mothers' knowledge was assessed using eight immune-related questions. Accordingly, mothers who answered four or more questions were assigned as having good knowledge, while those who answered less than four questions were assigned as having poor knowledge about immunization. Accordingly, 53 (63.9%) respondents categorized as cases and 157 (94.6%) respondents categorized as controls had good knowledge about immunization.

### Health facility and COVID-19-related characteristics

As reported by 64 (77.1%) cases and 151 (91%) controls, health facilities were found to be less than an hour's commute from their homes for vaccination services. The fear of COVID-19 to attend health facilities for vaccination was reported by most caretakers in 77(92.8%) cases and 153 (92.2%) controls, respectively. The health facility and COVID-19-related characteristics are shown in Table 3.

**Table 3:** Health facility and COVID-19 related characteristics of the respondents at Kacha Birra district, kembata Tembaro zone, south Ethiopia, 2022.

**Variables**	**Category**	**Immunization default**	
		**Cases [No. (%)]**	**Controls [No. (%)]**
Presence of nearby health facility	Yes	64 (77.1)	151 (91.0)
	No	19 (22.9)	15 (9.0)
Type of nearby health facility	Hospital	13 (15.7)	28 (16.9)
	Health center	9 (10.8)	19 (11.4)
	Health post	42 (50.6)	104 (62.7)
Distance to health facility (in walk time)	≤ 15 min	14 (16.9)	59 (35.5)
	15–30 min	29 (34.9)	66 (39.8)
	30–60 min	19 (22.9)	28 (16.9)
	>60 min	21 (25.3)	13 (7.8)
Postponing vaccine schedule	Yes	37 (44.6)	29 (17.5)
	No	46 (55.4)	137 (82.5)
Waiting time spent in the health facility	≤ 1 h	28 (33.7)	95 (57.2)
	1–2 h	41 (49.4)	48 (28.9)
	2–3 h	11 (13.3)	17 (10.2)
	>3 h	3 (3.6)	6 (3.6)
Ever tested for COVID-19	Yes	16 (19.3)	32 (19.3)
	No	67 (80.7)	134 (80.7)
Result of COVID-19 test	Positive	2 (12.5)	6 (18.8)
	Negative	14 (87.5)	26 (81.3)
Fear of COVID-19 to attend health facility	Yes	6 (7.2)	13 (7.8)
	No	77 (92.8)	153 (92.2)

#### Determinants of default from completion of child immunization

According to the results of the multivariable logistic regression analysis, several factors were found to be significantly associated with default from completion of child immunization. These factors include postponing the vaccine schedule, poor knowledge of mothers or caretakers about vaccination, parity greater than four, and no ANC follow-up. These findings were obtained after adjusting for all other variables.

The odds of immunization default were five times higher among children born to mothers who had no ANC follow-up compared to those born to mothers who had ANC follow-up [AOR = 5.40, 95% CI (2.24–13.52)]. The odds of immunization default were twice as high among children born to mothers who postponed vaccination schedules as those born to mothers who non-postponed vaccination schedules [AOR = 2.28, 95% CI: (1.05–4.93)].

The odds of immunization default among children born to mothers with a parity greater than four were three times higher compared to those born to mothers with a parity less than or equal to four [AOR = 3.25, 95% CI: (1.45–7.27)].

The odds of immunization default were 6.7 times higher among children born to mothers with poor knowledge of immunization compared to those born to mothers with good knowledge about immunization [AOR = 6.77, 95% CI: (2.33–19.64)]. The results of the multivariate analysis are presented in Table 4.

**Table 4:** Multivariable analysis of determinants of default from completion of childhood immunization among children 15–23 months old, Kacha Birra District, Kembata Tembaro zone, south Ethiopia, June 2022.

**Variables**	**Category**	**Immunization default**		**COR, 95%CI**	**AOR, 95%CI**	***P*-value**
		**Cases [No. (%)]**	**Controls [No. (%)]**			
Place of birth	Health institution	71 (85.5)	162 (97.6)	1	1	
	Home	12 (14.5)	4 (2.4)	6.85 (2.13–21.9)	2.56 (0.48–14.0)	0.270
Availability of vaccination card	Yes	64 (77.1)	148 (89.2)	1	1	
	No	19 (22.9)	18 (10.8)	2.44 (1.20–4.96)	1.60 (0.63–4.10)	0.321
ANC follow up	Yes	53 (63.9)	149 (89.8)	1	1	
	No	30 (36.1)	17 (10.2)	4.96 (2.53–9.72)	**5.40 (2.24–13.5)**	**< 0.001**
Received TT vaccine	Yes	50 (60.2)	132 (79.5)	1	1	
	No	33 (39.8)	34 (20.5)	2.56 (1.44–4.57)	1.19 (0.53–2.70)	0.673
PNC follow up	Yes	33 (39.8)	118 (71.1)	1	1	
	No	50 (60.2)	48 (28.9)	1.93 (1.13–3.29)	0.91 (0.43–1.94)	0.805
Presence of nearby health facility	Yes	64 (77.1)	151 (91.0)	1	1	
	No	19 (22.9)	15 (9.0)	2.99 (1.43–6.25)	0.63 (0.17–2.33)	0.482
Distance to health facility (in walk time)	≤ 15 min	14 (16.9)	59 (35.5)	1	1	
	15–30 min	29 (34.9)	66 (39.8)	1.85 (0.89–3.83)	1.5 (0.59–3.73)	0.393
	30–60 min	19 (22.9)	28 (16.9)	2.86 (1.25–6.50)	0.89 (0.28–2.79)	0.846
	>60 min	21 (25.3)	13 (7.8)	6.80 (2.76–16.8)	4.22 (0.96–18.5)	0.057
Postponing vaccine schedule	Yes	37 (44.6)	29 (17.5)	3.80 (2.11–6.85)	**2.28 (1.05–4.93)**	**0.037**
	No	46 (55.4)	137 (82.5)	1	1	
Waiting time spent in the health facility	≤ 1 h	28 (33.7)	95 (57.2)	1	1	
	1–2 h	41 (49.4)	48 (28.9)	2.89 (1.60–5.24)	1.75 (0.77–3.95)	0.178
	2–3 h	11 (13.3)	17 (10.2)	2.19 (0.92–5.23)	1.15 (0.37–3.59)	0.806
	>3 h	3 (3.6)	6 (3.6)	1.69 (0.39–7.22)	1.32 (0.19–9.06)	0.780
Parity	≤ 4	49 (66.2)	126 (80.8)	1	1	
	>4	25 (33.8)	30 (19.2)	2.14 (1.15–4.0)	**3.25 (1.45–7.27)**	**0.004**
Knowledge of mothers	Poor	30 (36.1)	9 (5.4)	9.87 (4.40–22.14)	**6.77 (2.33–19.6)**	**< 0.001**
	Good	53 (63.9)	157 (94.6)	1	1	

The bold values shows significantly associated variables.

## Discussion

This study assessed the determinants of defaulting from completion of immunization among children aged 15–23 months living in the Kacha Bira district of South Ethiopia. The study found that the absence of antenatal care follow-up, postponement of the vaccination schedule, having more than four children, and poor knowledge of mothers about immunization were the main reasons for defaulting from completion of immunization among children aged 15–23 months.

Failure to receive ANC follow-up was a determinant of default from completion of child immunization. The odds of defaulting from completion of immunization among children born to mothers who had no ANC follow-up was five times higher compared to those born to mothers who had ANC follow-up. This is in line with the studies conducted in Ethiopia, Senegal, Nigeria, and the Wonago district of South Ethiopia (21, 23–25). This finding was not surprising, given that attending antenatal care during pregnancy creates opportunities for pregnant mothers to obtain adequate information on immunization and vaccine-preventable diseases.

Our findings also showed that postponing the vaccination schedule was a factor that determined defaulting from immunization. This finding was similar to those studies conducted in Malaysia, Gambella district in Southwest Ethiopia, and Arbegona district in South Ethiopia (26–28). A possible reason could be related to the accessibility of health facilities, vaccine stock-outs in the facility, forgetting the schedule of immunization, and long waiting times in the facility. This is because, in developing countries such as Ethiopia, accessibility of health facilities and vaccines is very challenging, especially in rural areas. In addition, caretakers/mothers often forget their appointments for vaccination.

The findings of this study also revealed that the parity of the mother is a determinant of immunization defaulting. The odds of defaulting from immunization for mothers with parity greater than four were three times higher compared to others. This finding is supported by the studies conducted in Bangladesh, Kenya, and the Ginir district of southeastern Ethiopia (17, 29–31). This might be because having more children may lead to resource constraints, and as the number of children is higher, the cost and demand for resources become higher, which has a negative impact on healthcare utilization.

Maternal knowledge about immunization was another factor that determined defaulting from immunization. The odds of defaulting from completion of immunization were seven times higher for mothers (caretakers) who had poor knowledge of immunization compared to those with good knowledge about immunization. This finding can be explained by the fact that mothers who have good knowledge about immunization may have better attitudes about the benefit of child immunization than mothers who have poor knowledge. The finding was consistent with the study conducted here in Ethiopia (20), Nepal, Nigeria, and Southwest Ethiopia (19, 20, 32, 33). A possible explanation for this could be that mothers (caretakers) who had good knowledge of immunization were encouraged more to immunize their children regularly than mothers (caretakers) who had poor knowledge of immunization.

### Limitation

Recall bias may occur if mothers forget details about their children's vaccinations, including the number of vaccine doses received and other related information.

## Conclusion and recommendation

This study aimed at identifying the factors that contribute to the failure of completing immunization among children aged 15–23 months. The study found that a lack of antenatal care follow-up, postponing vaccination schedule, mothers with more than four children, and inadequate knowledge of mothers about immunization were significant determinants of defaulting from completing immunization among children aged 15–23 months.

Based on the findings of this study, we offer the following recommendations:

✓ Health providers should motivate and counsel mothers to attend ANC at health facilities.
✓ Healthcare workers and health administering bodies should work together to avoid the problems that cause postponing vaccination schedules.

## Data availability statement

The original contributions presented in the study are included in the article/supplementary material, further inquiries can be directed to the corresponding author.

## Ethics statement

The study was reviewed and approved by the Institutional Review Board of Wolaita Sodo University at the College of Health Sciences and Medicine (CHSM/ERC/01/14). Besides, written informed consent for participation was obtained for all study participants.

## Author contributions

SM: Conceptualization, Formal analysis, Project administration, Visualization, Writing—original draft. EW: Data curation, Methodology, Supervision, Validation, Visualization, Writing—review & editing. SK: Data curation, Methodology, Supervision, Validation, Visualization, Writing—review & editing. MU: Software, Writing—review & editing. SH: Software, Writing—review & editing. DL: Software, Writing—review & editing. EA: Writing—original draft, Writing—review & editing, Methodology, Validation, Software. EB: Formal analysis, Methodology, Software, Visualization, Writing—review & editing.
